# Prevalence of HIV, Herpes Simplex Virus-2, and Syphilis in male sex partners of pregnant women in Peru

**DOI:** 10.1186/1471-2458-8-65

**Published:** 2008-02-19

**Authors:** Jesse L Clark, Kelika A Konda, Cesar V Munayco, Monica Pún, Andres G Lescano, Segundo R Leon, Jose Pajuelo, Luis Suarez-Ognio, Jeffrey D Klausner, Thomas J Coates, Carlos F Cáceres

**Affiliations:** 1 University of California, Los Angeles, California, USA; 2 Dirección General de Epidemiología, Lima, Peru; 3 Universidad Peruana Cayetano Heredia: Lima, Peru; 4 San Francisco Department of Public Health, San Francisco, California, USA

## Abstract

**Background:**

Sexually active heterosexual men may represent an important risk factor for HIV infection and STI transmission to their female partners and unborn children, though little is known about the prevalence of STIs in this population. We sought to determine the prevalence of HIV, herpes simplex virus type 2 (HSV-2), and syphilis infection and associated risk behaviors among male sex partners of pregnant women in Peru.

**Methods:**

Survey and seroprevalence data were collected from 1,835 male partners of pregnant women in four cities in Peru. Serum was tested for antibodies to HIV, HSV-2, and syphilis.

**Results:**

Among the 1,835 male participants, HIV prevalence was 0.8% (95% CI = 0.5–1.4%), HSV-2 16.0% (95% CI = 14.3–17.8%), and syphilis 1.6% (95% CI = 1.0–2.2%). Additionally, 11.0% reported a lifetime history of intercourse with men, and 37.1% with female sex workers. Unprotected intercourse with men during the previous year was reported by 0.9% and with female sex workers by 1.2%.

**Conclusion:**

Pregnant women's sex partners reported lifetime sexual contact with core risk groups, had an elevated prevalence of HSV-2, and demonstrated the potential to spread HIV and other STIs to their partners. Though the prevalence of HIV in the population was not significantly higher than observed in other samples of heterosexuals in Peru, the risk of HIV transmission to their female partners may be exacerbated by their increased prevalence of HSV-2 infection. Further study of heterosexual populations is necessary to fully understand the epidemiology of HIV/STIs in Latin America.

## Background

The HIV epidemic in Peru remains concentrated in the core risk group of men who have sex with men (MSM) without extension into the general population [1-3]. While research has been conducted on the social and biological mechanisms of disease transmission between MSM in the region, little attention has been paid to the issue of HIV infection and sexually transmitted infections (STIs) among heterosexuals in Peru [4-6]. There is a need to further understand the epidemiology of HIV infection and STIs in Peru's general population in order to assess the potential for the spread of HIV/STIs within heterosexual networks.

Research has suggested that men in Peru generally have greater numbers of sex partners, and greater risk of potential STI exposures, than women [7,8]. In studies of pregnant women in Lima, the sexual behavior of male partners was an important factor both in increasing the size of women's sexual networks, and in establishing women's indirect exposure to high-risk communities [9]. Among HIV-infected pregnant women, 26.7% of their HIV-positive male partners had engaged in sexual contact with other men and 46.7% had engaged in unprotected sex with a female sex worker. Yet, as in most other HIV surveillance studies in Peru, HIV prevalence in the study population was less than 1%, suggesting that the primary impact of male partners' risk behavior was on individual risk for infection without significantly impacting the population-level spread of disease.

Other analyses have investigated STIs and associated risk behaviors in subgroups of high-risk heterosexually active men in Peru, but few recent studies have examined the prevalence of disease or high-risk behavior within the general heterosexual population. In one study of male clients of female sex workers in Peru, chlamydial and gonococcal infections were uncommon, and only 4.2% of men surveyed reported engaging in unprotected intercourse with female sex workers [10]. In a separate study, men sexually active with both men and women had an elevated prevalence of HIV infection (11.1%), and high rates of unprotected vaginal and anal intercourse with both male and female partners [11]. In another analysis of heterosexual-identified men in low-income urban neighborhoods in Peru, 14.2% reported recent male sex partners, with the majority of those men engaging in unprotected sex with both male and female partners (84.2% and 57.0%, respectively) [12]. Additionally, a survey of heterosexual couples seeking treatment at STI clinics in Lima found frequent reports of risk behaviors such as unprotected sex with casual partners, male same-sex contact, and sex with female sex workers [6]. While these studies defined the risk behaviors of selected high-risk sub-groups, they did not estimate the size of these communities as a proportion of the overall population. In contrast, a 1991 survey of Peru's general population assessed HIV/STI prevalence and associated risk factors, and found high prevalences of reported risk behavior among male participants [8]. In a sample of 600 men and women, men reported ten times as many sex partners as women, and 36.6% of men reported contact with female sex workers. Only 8.9% of men reported always using condoms during intercourse with casual partners. However, only 7.7% of the men were found to have antibodies to HSV-2 and only one case of HIV infection was diagnosed in the study population. No recent studies have reported prevalence of HIV/STIs or associated risk factors for transmission among adults in the general population. We studied the prevalence of HIV infection and STIs among male partners of pregnant women in Peru to determine the prevalence of infections among heterosexually active males, and to assess their potential role in HIV/STI transmission to pregnant women and neonates in Peru.

## Methods

### Study Design, Population and Recruitment

We conducted a cross-sectional study of prevalence and risk factors for STIs among the male partners of women attending prenatal and post-partum visits at 43 health centers in Peru. The study was performed in the coastal cities of Lima, Ica, Trujillo, and Chiclayo, capitol cities of provinces reporting some of the highest numbers of HIV infections in Peru [2]. Of the 16,888 cases of HIV infection diagnosed in Peru between 1983 and 2004, 73.1% were reported in the provinces of Lima/Callao, 3.1% in Ica, 2.3% in La Libertad (Trujillo), and 1.6% in Lambayeque (Chiclayo). Other provinces individually contributed between 0.2% and 2.6% of the total number of HIV infections diagnosed in Peru. Eligible participants included adult male sex partners of women attending the selected health centers for prenatal or post-partum services between December 2003 and February 2004. All eligible subjects agreeing to participate provided written informed consent. The Committee of Human Subjects Research of the University of California at Los Angeles (UCLA), the University of California at San Francisco (UCSF), the Universidad Peruana Cayetano Heredia (UPCH), and the respective regional authorities of the Peruvian Ministry of Health approved the study.

We projected a sample of 2,062 participants, assuming a distribution across cities proportional to the total population of each city. Candidates were adult men who either accompanied pregnant women to prenatal visits, were present during a prenatal home visit, visited women who had recently given birth in the hospital, or accompanied women with infants less than 48 hours old to the initial newborn check-up. Potential participants were informed of the study by the obstetrician caring for the man's pregnant partner. Men identified during prenatal home visits were informed of the study by their partner's obstetrician and asked to present themselves to the study site if they wished to enroll. Anyone who denied a history of sexual contact with the pregnant woman was excluded from the study.

### Data Collection

Participants were interviewed in private within the health center by the obstetrician caring for the subject's partner using a structured questionnaire. The survey collected information regarding sociodemographic factors, sexual history, sexual risk behaviors, and substance use. For the purposes of data collection, "recent" sexual contact was defined as sexual contact within the preceding 12 months. A 10-mL blood sample was collected from all participants and assigned an identification code consisting of the participant's initials and date of birth, as required by Peru's Ministry of Health, to track newly diagnosed cases of HIV infection.

### Laboratory Methods

All blood samples were tested in the respective onsite hospital laboratories for syphilis and HIV infection by RPR and ELISA screening. Herpes Simplex Virus-2 (HSV-2) ELISA and HIV Western Blot assays were performed at the central laboratory at Universidad Peruana Cayetano Heredia. Syphilis screening was performed by RPR assay (RPRnosticon, Biomérieux; Marcy l'Étoile, France) with TPPA confirmation of positive results (Serodia, Fujirebio; Tokyo, Japan). Two HIV ELISAs were used for screening (Vironostika, bioMérieux; Marcy l'Étoile, France; Genetic Systems, Biorad; Hercules, CA, USA) and samples positive by one or both ELISA assays were confirmed by Western Blot (Genetic Systems, Biorad; Hercules, CA, USA). Inconclusive results on the Western Blot assay were interpreted as negative. HSV-2 infection was determined by HSV-2 specific ELISA (HerpeSelect, Focus Technologies; Cypress, CA, USA) using a cut-off value of ≤ 1.1 to define seropositivity.

Participants were instructed to return to the study site 10 days after sample collection for post-test counseling and provision of test results. Men with serologic evidence of syphilis infection were treated according to Peruvian Ministry of Health guidelines with 2.4 million units benzathine penicillin G intramuscularly once and then monitored for response to treatment by repeated serologic testing at 3, 6, and 9 months. Those participants diagnosed with HIV infection were referred to local facilities designated by the Ministry of Health for ongoing management of HIV infection, including antiretroviral therapy when appropriate. Participants diagnosed with HSV-2 infection by serology were not provided with antiviral treatment unless they reported an active herpes outbreak, in which case they were provided with Acyclovir 200 mg orally 5 times daily for a period of 10 days. All participants diagnosed with an STI were advised of the importance of partner notification.

### Data Analysis

HIV, HSV-2, and syphilis prevalence were the primary outcomes of interest and were analyzed as dichotomous variables. The association of STIs and potential covariates were analyzed using contingency tables and logistic regression to determine differences in STI prevalence. Odds-ratios were estimated for potential risk factors for each outcome separately. In multivariate regression for HSV-2 and syphilis seropositivity, variables were selected by sequentially adding the most significant predictors determined from the bivariate analysis into the model in a nested fashion. Likelihood ratios were used to determine the statistical significance of the associations. No multivariate regression analysis was possible for HIV infection due to the small number of HIV-positive subjects. Stata 8.0 software was used for all analyses (Stata Corporation, College Station, TX).

## Results

### Enrollment

Between December 15, 2003 and January 24, 2004, 1,835 participants were enrolled, with acceptance rates ranging between 80 to 90% for the different sites: 1439 subjects were enrolled in Lima/Callao, 186 in Trujillo, 107 in Chiclayo, and 103 in Ica. The distribution of subjects in the study sample was proportionate to population size of the respective region by a margin of +/- 3%. HIV results were available for 1834 participants, HSV-2 for 1797, and syphilis for 1835.

### Sociodemographic Characteristics

Table 1 shows participant demographic characteristics. Most participants had at least some secondary school education (90.2%). The vast majority (93.0%) described the pregnant woman as their primary partner ("live-in partner" or "wife") and 72.1% had been in a relationship with the woman for one or more years.

**Table 1:** Select Demographic Characteristics of Male Sex Partners of Pregnant Women; Peru, 2003–2004*.

**Characteristics**		**N**	**Percent**
**Age (Years) (Mean ± s.d.)**		28.5 ± 7.4	
**Education**			
	**Primary or less**	175	9.8
	**High school or less**	1392	77.5
	**Some higher education**	228	12.7
**City**			
	**Lima/Callao**	1400	77.9
	**Trujillo**	186	10.4
	**Chiclayo**	107	6.0
	**Ica**	103	5.7
**Employment**			
	**Steady employment**	977	54.1
	**Occasional employment**	403	22.5
	**Unemployed**	82	4.6
	**Other**	335	18.7
**Relationship w/pregnant woman**			
	**Girlfriend/Fiancée**	124	7.0
	**Live-in partner**	1228	68.9
	**Wife**	431	24.1
**Time with pregnant woman (years, Median IQR)**		2 (1–5)	
**Lifetime sexual partners (Median IQR)**		4 (2–8)	
**Sexually active years (Mean ± s.d.)**		12.2 ± 7.9	
**Age at sexual debut (years) (Mean ± s.d.)**		16.3 ± 3.3	
**Previous STI**		207	11.5
**Genital ulcer, last 12 months**		204	11.4
**(Non-injection) Drug use, last 12 months**		125	7.0
**Sexual contact with FSW, lifetime**		668	37.1
**Sexual contact with FSW, previous 12 months**		158	8.9
**Unprotected sexual contact with FSW, previous 12 months**		22	1.2
**Sexual contact with male, lifetime**		197	11.0
**Sexual contact with male, previous 12 months**		31	1.7
**Unprotected sexual contact with male, previous 12 months**		16	0.9

* Complete survey response data not available for some participant

### Prevalence of STIs

The prevalence of HIV infection in the study population was 0.8% (n/N = 15/1834; 95% Confidence Interval [CI] = 0.5 – 1.4%), HSV-2 prevalence was 16.0% (287/1797; 95% CI = 14.3–17.8%) and syphilis prevalence was 1.5% (28/1835; 95% CI = 1.0–2.2%) (Table 2).

**Table 2:** Factors Associated with HSV-2, HIV and Syphilis Infection Among Male Sex Partners of Pregnant Women; Peru, 2003–2004.

Risk Factor	HSV-2 n/N (%)	Syphilis n/N (%)	HIV n/N (%)
Overall	287/1796 (15.9%)	28/1796 (1.6%)	15/1796 (0.8%)
Age			
16 – 22	37/383 (9.7%)	5/383 (1.3%)	2/383 (0.5%)
23 – 26	51/462 (11.0%)	3/462 (0.6%)	6/462 (1.3%)
27 – 32	85/467 (17.5%)	9/487 (1.8%)	4/487 (0.8%)
33+	114/463 (24.6%)^‡^	11/463 (2.4%)	3/463 (0.6%)
# Lifetime sexual partners			
1 – 2	42/362 (11.6%)	3/362 (0.8%)	2/362 (0.6%)
3 – 4	66/435 (15.2%)	2/435 (0.5%)	3/435 (0.7%)
5 – 9	57/429 (13.3%)	9/429 (2.1%)	3/429 (0.7%)
10+	108/432 (25.0%)^‡^	11/432 (2.5%)*	6/432 (1.4%)
# Sexually active years			
0 – 6	39/450 (8.7%)	4/450 (0.9%)	3/450 (0.7%)
7 – 11	59/522 (11.3%)	6/522 (1.1%)	4/522 (0.8%)
12 – 17	80/416 (19.2%)	7/416 (1.7%)	5/416 (1.2%)
18+	109/407 (26.8%)^‡^	11/407 (2.7%)*	3/407 (0.7%)
History of STI			
No	226/1589 (14.2%)	20/1589 (1.3%)	11/1589 (0.7%)
Yes	61/204 (29.9%)‡	8/207 (3.9%)^†^	4/207 (1.9%)
History of sex with men, lifetime			
No	143/1599 (14.6%)	23/1599 (1.4%)	12/1599 (0.8%)
Yes	54/197 (27.4%)^‡^	5/197 (2.5%)	3/197 (1.5%)
History of sex with FSW, lifetime			
No	150/1128 (13.3%)	15/1128 (1.3%)	10/1128 (0.9%)
Yes	137/688 (20.5%)^‡^	13/668 (1.9%)	5/668 (0.7%)
Non-injection illegal drug use, last year			
No	256/1671 (15.3%)	26/1671 (1.6%)	13/1671 (0.8%)
Yes	31/125 (24.8%)^†^	2/125 (1.6%)	2/125 (1.6%)
Genital ulcer, last year			
No	226/1592 (14.2%)	19/1592 (1.2%)	13/1592 (0.8%)
Yes	61/204 (29.9%)‡	9/204 (4.4%)^‡^	2/204 (1.0%)

*<0.05 †<0.01 ‡ <0.001, determined by chi-squared tests and tests of trend for categorized continuous variables (age, number of lifetime partners, and number of sexually active years)

### Sexual Behavior

The mean number of lifetime sex partners among participants was 9.9 (standard deviation [s.d.] +/- 35.1) and mean age at sexual debut was 16.3 (s.d. +/- 3.3) years. While all participants reported sexual contact with the pregnant woman within the past year as a condition of enrollment in the study, 94.1% reported unprotected intercourse with their pregnant partner in the month prior to conception. Previous sexual contact with a female sex worker during their lifetime was reported by 37.1% of participants while 8.9% reported female sex worker contact during the past year and 1.2% had unprotected intercourse with a female sex worker during that time. Previous sexual contact with another man was reported by 11.0% of subjects, though only 1.7% of all participants reported male sexual contacts in the previous year and 0.9% of the total sample described unprotected sex with men during that time. Participants were less likely to have reported sexual contact in the past year with other men (1.7%) than with female sex workers (8.9%, p = 0.001). Of the 31 participants reporting sexual contact with men in the past year, 51.6% stated they had unprotected intercourse during these encounters and 45.2% reported recent contact with female sex workers, suggesting a concentration of behavioral risk factors within this small subgroup.

### Risk Factors Associated with STIs

In bivariate logistic regression, HIV seropositivity was associated with HSV-2 infection (Odds Ratio [OR] = 8.12; p < 0.001) (Table 3). In multiple logistic regression, HSV-2 infection was significantly associated with additional years of sexual activity (Adjusted OR (aOR) = 1.07; p < 0.001), HIV infection (aOR = 8.41; p < 0.001), and recent illegal drug use (aOR = 1.86; p = 0.008). In multiple logistic regression, syphilis infection was associated with an increased number of sexual partners (aOR = 1.01; p = 0.022). There was no difference in condom use according to STI diagnosis (all p-values >0.05).

**Table 3:** Factors Associated with HSV-2, HIV and Syphilis Infection Among Male Sex Partners of Pregnant Women; Peru, 2003–2004.

Risk Factor	HSV-2		Syphilis		HIV Unadjusted OR (95% CI)
	Unadjusted OR (95% CI)	Adjusted OR (p-value, 95% CI)	Unadjusted OR (95% CI)	Adjusted OR (95% CI)	
Age (per additional year)	1.07^‡ ^(1.05 – 1.08)	-	1.06^† ^(1.02 – 1.10)	1.04 (1.00 – 1.09)	1.00 0.93 – 1.07
# Lifetime sexual partners (per additional partner)	1.01^† ^(1.00 – 1.01)	-	1.01^† ^(1.00 – 1.01)	1.00 (1.00 – 1.01)	1.00 (0.97 – 1.02)
# Sexually active years (per additional year)	1.07^‡ ^(1.05 – 1.08)	1.07 (<0.001, 1.05 – 1.09)	1.06^† ^(1.02 – 1.10)	-	1.00 (0.94 – 1.07)
History of sex with men, lifetime	2.22^‡ ^(1.57 – 3.11)	-	1.78 (0.67 – 4.75)	-	2.06 (0.57 – 7.31)
History of sex with FSW, lifetime	1.68^‡ ^(1.30 – 2.17)	-	1.47 (0.70 – 3.11)		0.85 (0.29 – 2.48)
Non-injection illegal drug use, last year	1.82^† ^(1.19 – 2.79)	2.23 (0.008, 1.43 – 3.49)	1.03 (0.24 – 4.39)	-	2.08 (0.46 – 9.29)

HSV-2 infection	-	-	3.50^† ^(1.62 – 7.55)	2.09 (0.89 – 4.91)	8.12^‡ ^(2.86 – 22.96)
Syphilis infection	3.50^† ^(1.62 – 7.55)	-	-	-	N/A°
HIV infection	8.12^‡ ^(2.86 – 22.96)	8.58 (<0.001, 2.95 – 24.99)	N/A°	N/A°	-

*<0.05 †<0.01 ‡ <0.001

°Values could not be determined due to the absence of cases of HIV/Syphilis co-infection in the study population.

## Discussion

Our results present an epidemiologic portrait of STI prevalence and risk behavior among male partners of pregnant women in coastal Peru. The rate of HSV-2 infection among men in our study indicates an important risk factor for HIV infection and potential congenital herpes transmission for HSV-2 naive female partners. In addition, a minority of men (less than 1.0%) had recent high-risk secondary sexual partnerships, indicating potential increase in risk for new HIV/STI acquisition and subsequent transmission to their partners and newborn children.

The 0.8% prevalence of HIV infection among our subjects was slightly higher than previous HIV surveillance estimates of 0.3–0.6% among pregnant women [3] and 0.4% among general population males [13]. However, it is not clear if the slightly elevated HIV prevalence reflects a significant increase in HIV infections in this population, or a statistical variance within in our sample. Additional studies are needed to address this issue.

The percentage of subjects with serologic evidence of HSV-2 infection (16.0%) suggests that genital ulcerative disease may be an important risk factor for HIV infection in this community. The link between genital herpes and HIV infection has been well established [14], with recently published studies specifically addressing the importance of HSV-2 seropositivity as a risk factor for HIV transmission in MSM populations in Peru [15,16]. The presence of HSV-2 infection indicates a potential mechanism for future HIV transmission, despite inconclusive findings concerning prevalence of HIV infection in this population. In addition, the high risk for genital herpes transmission to HSV-2 negative pregnant women from their HSV-2 positive male partners speaks to the need for improved public health control of HSV-2, including educational campaigns to decrease transmission by limiting sexual contact and increasing use of antiviral treatment (e.g., acyclovir) during active outbreaks.

Our study has methodological limitations that may have influenced our findings. Interviews were conducted by the partner's obstetrician, which could have limited subjects' willingness to report recent sex partners outside of their current relationship, though reported lifetime contacts with MSM and female sex workers are consistent with other published studies [6,8,13]. A further complication is the fact that the majority of study participants (70%) were enrolled at hospital recruitment sites, with only 30% enrolled during home visits. This discrepancy in recruitment is probably due to the fact that participants recruited during home visits were obligated to attend an additional enrollment visit at the hospital study site in order to participate. As a result men less committed to the pregnancy of their partner, and possibly more likely to engage in sexual risk behavior outside of the relationship, were less likely to be included in the study. The refusal rates of participants recruited at the different sites were not recorded. Although men in our study are a sample of heterosexually active men engaging in unprotected intercourse, their status as the partner of a pregnant woman is also likely to have modified their risk behavior. Men outside of a committed relationship are likely to be at even higher risk for STIs and merit further study. Further, modifications in sexual practices that often occur as a result of a woman's pregnancy may also have substantially altered the risk behavior of one or both members of the partnership during the year preceding evaluation, significantly influencing the recent risk behaviors assessed in our study. As public health authorities consider the appropriateness of monitoring seroprevalence among pregnant women as a marker for disease prevalence in the general population, it may also be important to take steps to define an appropriate, corresponding male population for epidemiologic surveillance.

## Conclusion

Male partners of pregnant women in Peru reported previous contact with groups at high risk for infection with HIV and other STIs, especially female sex workers, though recent contact with high-risk populations and diagnosis of active STIs was limited. While the prevalence of HIV and syphilis infection was relatively low, the high rate of HSV-2 seropositivity indicates a cofactor for HIV acquisition and transmission and a potential source of neonatal herpes infection that should be addressed. A small subset of these men reported recent, high-risk contact with core risk groups including female sex workers and MSM, and present a higher level of risk for HIV/STI transmission to their female partners and infant children on an individual level. Consistent public health interventions are needed in Peru to reduce risks of STI acquisition through reducing the number of sex partners and increasing condom use among heterosexuals, as well as increasing awareness, availability and uptake of STI screening. Surveillance of HIV infection and STIs among the male partners of pregnant women is important to develop a comprehensive framework for the analysis of sexual health in Latin America.

## Competing interests

The author(s) declare that they have no competing interests.

## Authors' contributions

JLC analyzed study data and was the primary author of the manuscript; KAK analyzed study data and participated in revision of the manuscript; CVM conducted data acquisition; MP supervised data acquisition, AGL analyzed data and participated in revision of the manuscript; SRL supervised collection and analysis of biological specimens; JP participated in the development of the study concept; LS supervised data acquisition; JDK supervised data analysis and participated in revision of the manuscript; TJC supervised development of the study concept and design, supervised analysis of data, and participated in revision of the manuscript; CFC supervised development of the study concept and analysis of data and participated in revision of the manuscript. All authors read and approved the final version of the manuscript.

## Pre-publication history

The pre-publication history for this paper can be accessed here:


